# Sandwich-like layer-by-layer assembly of gold nanoparticles with tunable SERS properties

**DOI:** 10.3762/bjnano.7.95

**Published:** 2016-07-15

**Authors:** Zhicheng Liu, Lu Bai, Guizhe Zhao, Yaqing Liu

**Affiliations:** 1 Shanxi Province Key Laboratory of Functional Nanocomposites, School of Materials Science and Engineering, North University of China, Taiyuan 030051, China; 2 School of Chemical and Environmental Engineering, North University of China, Taiyuan 030051, China

**Keywords:** assembly, layer-by-layer, multilayer thin film, nanoparticle, polyelectrolyte

## Abstract

Sandwich-like layer-by-layer thin films consisting of polyelectrolytes and gold nanoparticles were utilized to construct surface-enhanced Raman scattering (SERS) substrates with tunable SERS properties. It is found that both the size of the nanoparticles in the layers and the interlayer distance significantly influence the SERS performance of the multilayered thin film. These simple, low-cost, easily processable and controllable SERS substrates have a promising future in the field of molecular sensing.

## Introduction

Surface-enhanced Raman scattering (SERS) spectroscopy, which relies on metal nanostructures made of noble metals (Au, Ag and Cu) that sustain localized surface plasmon resonance (LSPR), is applied as a promising analytical tool for detecting and identifying trace amounts of molecular species [1–3]. The fabrication of excellent SERS substrates using simple and low-cost methods is currently an attractive topic in this field [4]. Engineering metal nanoparticle assemblies with tunable plasmonic coupling properties shows high potential for that purpose [5].

Among various top-down and bottom-up techniques, layer-by-layer (LbL) assembly is a facile and cost-efficient way for the controllable deposition of numerous components [6–8]. Multilayer nanostructures with complex morphologies and functions could be prepared conveniently through the LbL assembly process, which is mainly driven by electrostatic interaction. Especially, multilayer thin films consisting of polymers and metal nanoparticles (NPs) have been extensively explored, and show interesting optical and SERS properties [9–14]. Wang and Dong et al. reported that polyelectrolyte–gold nanorod multilayer thin films could be obtained using LbL assembly techniques via electrostatic interactions [15]. By controlling the number of deposition layers, the plasmonic properties as well as the SERS properties could be tuned easily. Moreover, Kim and co-workers showed that gold nanoparticles (Au NPs) could be assembled onto polyelectrolyte multilayer films and act as seeds for the following NP growth [16]. The grown NP films were demonstrated to be stable and reproducible SERS substrates. In addition to assembling only one type of NPs, Zhang et al. fabricated bimetallic gold–silver multilayer films by alternating the adsorption of polyethyleneimine–silver ions and Au NPs onto substrates and the subsequent in situ reduction of the silver ions [17]. Compared with the parallel samples, the bimetallic LbL film showed improved SERS properties. Although a few examples of SERS substrates based on LbL strategy have been given, the design and engineering of such SERS substrates is still challenging, and the probing of the structure-dependent SERS performance remains a considerable issue.

Recently, we have shown that highly reproducible and stable SERS substrates could be obtained via LbL assembly of polyelectrolyte and Au NPs [18]. The tuning of SERS intensities was realized by varying the number of deposited Au NP layer. Here, we present that SERS properties of LbL thin film could be controlled by assembling Au NPs of different sizes or changing the interlayer distance between Au NP layers. Sandwich-like LbL thin films with three bilayers, which were fabricated by alternating deposition of polyelectrolytes and Au NPs, are designed to explore the relationship between multilayer nanostructures and SERS performance.

## Experimental

All chemicals such as poly(diallyldimethylammonium chloride) (PDDA, *M*_w_ = 200,000–350,000), poly(sodium 4-styrenesulfonate) (PSS, *M*_w_ = 70000), and 4-aminothiophenol (4-ATP) were obtained from Sigma-Aldrich and used without further treatment. Negatively charged citrate-stabilized Au NPs were prepared using the classic Turkevich method [19–21]. Briefly, a 50 mL aqueous solution that contained 0.5 mL 1 wt % aqueous HAuCl_4_ solution was heated to boil under gentle stirring. Then, a certain amount of 1 wt % sodium citrate solution was added quickly. The obtained red-wine colored NP solution was stored at 4 °C and used for the LbL assembly. Hydrophilic quartz slides were used to deposit the first PDDA layer. Sandwich-like LbL thin films were obtained by alternative immersion of PDDA (1 mg/mL, with 1.5 M NaCl, 30 min) and Au NP solution (12 h). After each immersion step, the thin film was rinsed with water and dried under N_2_. Since only two different sizes of Au NPs were utilized, four kinds of sandwich-like LbL thin films consisting of NP layers of different NP sizes were achieved. The LbL thin film with three layers of small Au NPs is marked as SSS, while the one with three layers of big Au NPs is marked as BBB. Similarly, two other kinds of thin films, namely SBS and BSB, were also obtained. In order to control the interlayer distance between the Au NP layers, different numbers of PDDA/PSS bilayers were inserted into the sandwich-like nanostructure to separate the NP layers. Before SERS measurements, 50 μL of 0.1 mM 4-ATP ethanol solution was dropped on the as-prepared substrate and left to dry in the air.

The as-synthesized Au NP solution was characterized by UV–visible (UV–vis) spectroscopy (Cary 5000). Field emission scanning electron microscopy (FE-SEM, Hitachi S-4800) and transmission electron microscopy (TEM, JEOL JEM 1011) were used to image the LbL thin films and the NPs, respectively. SERS spectra excited at 1064 nm were recorded with a Thermo Nicolet FT-Raman 960 spectrometer at a power of 200 mW. All the spectra were collected by averaging 1024 scans.

## Results and Discussion

**Figure 1:**
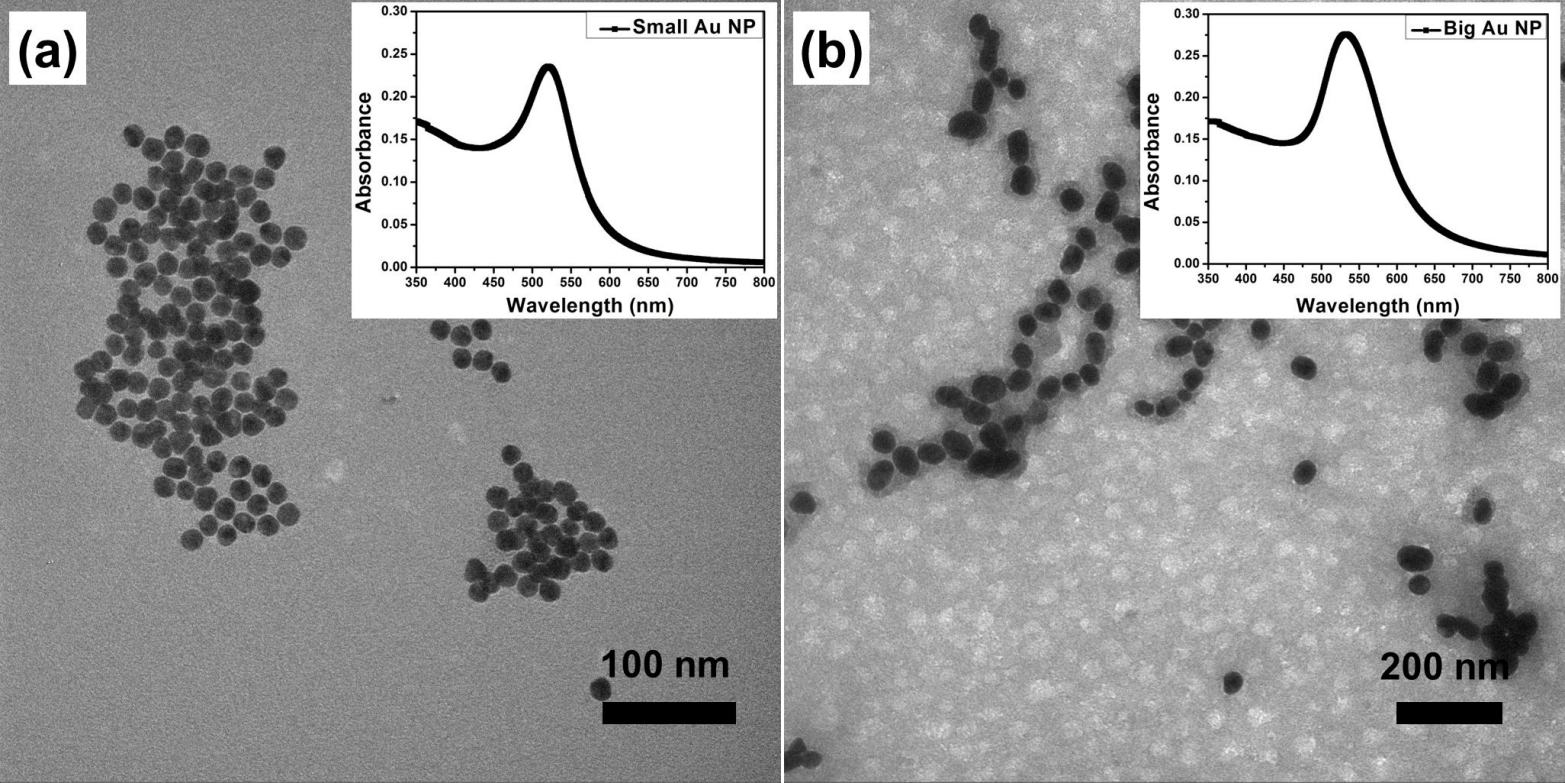
TEM images of the obtained Au NPs: (a) small Au NP, (b) big Au NP. The insets show the UV–vis spectra of the corresponding Au NP solutions.

Au NPs are regarded as certified and promising nanoscale building blocks for both LbL assembly and SERS substrates. Two different sizes of Au NPs were synthesized by simple control of the added volume of sodium citrate solution. Small Au NPs (17.0 ± 1.2 nm) were obtained when 2 mL sodium citrate solution was injected, while big Au NPs (42.9 ± 5.4 nm) were obtained when 0.65 mL sodium citrate solution was injected, as shown in Figure 1. The optical absorption peaks located at 520 and 532 nm indicate the strong surface plasmon resonance of the NPs. These uniform negatively charged citrate-protected Au NPs could be readily used in electrostatic LbL assembly.

**Figure 2:**
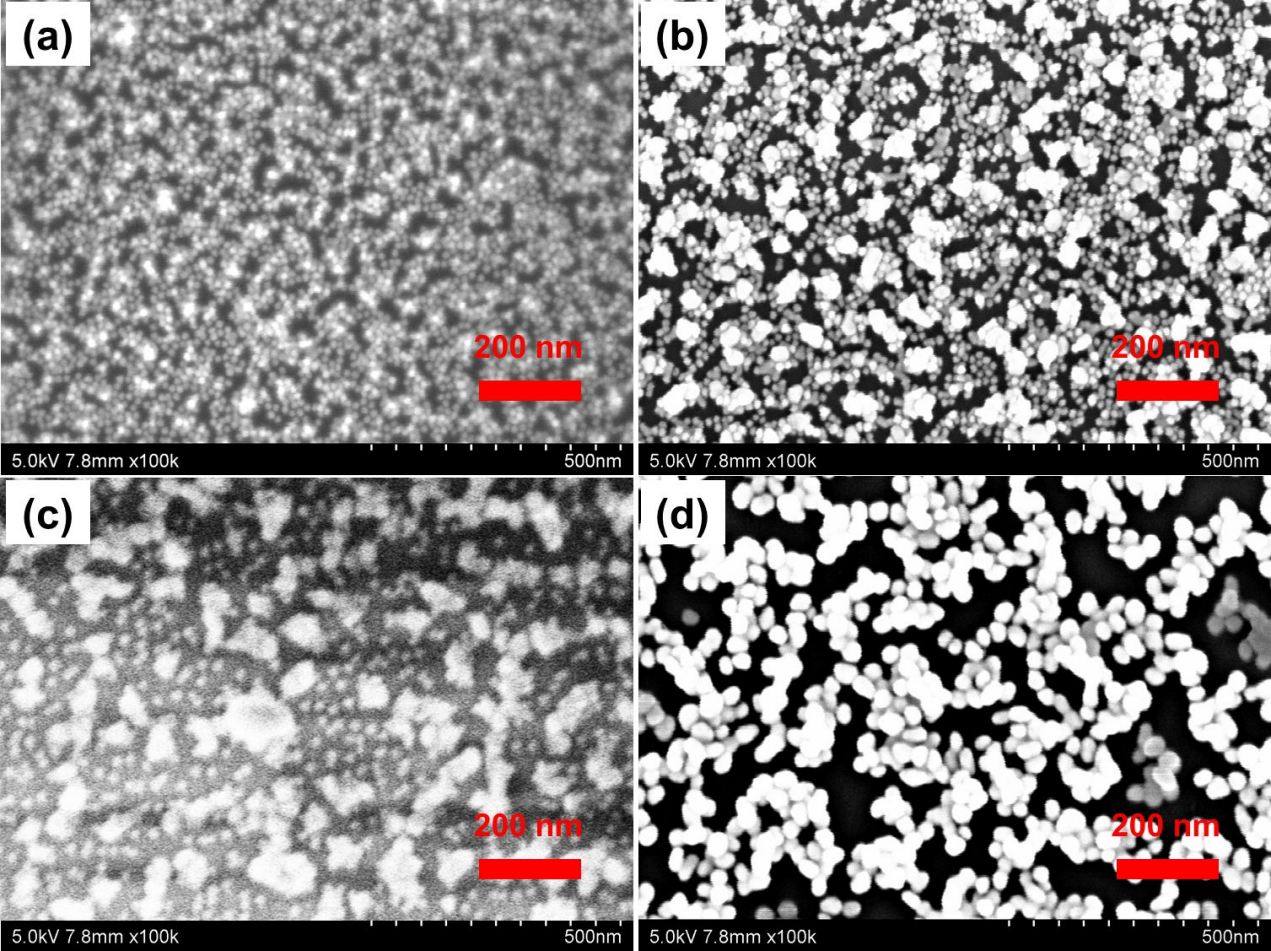
SEM images of multilayer thin films consisted of PDDA and Au NPs of different sizes: (a) SSS, (b) SBS, (c) BSB, (d) BBB.

Typical sandwich-like multilayer thin films were prepared by sequential deposition of polyelectrolytes and Au NPs. For example, the BSB thin film formed after the substrate was alternately dipped into PDDA/big Au NP/PDDA/small Au NP/PDDA/big Au NP solutions. From the point of view of Au NPs, these sandwich-like nanostructures are ideal for the evaluation of NP size effects on the SERS performance of NP assemblies. Figure 2 presents the SEM images of the SSS, SBS, BSB and BBB multilayer thin films. Undoubtedly, both small and big Au NPs were assembled into the thin films, and the NP size did not change during the assembly process. It is clear that the sandwich-like LbL assembly of Au NPs occurs via lateral expansion mode, which is consistent with previous results [18,22–23]. Interestingly, compared with the BSB thin film, there were more small Au NPs in the SBS thin film, though the number of big Au NPs was about the same. This phenomenon may result from the steric hindrance of the pre-assembled big Au NPs. In other words, the first big NP layer in the BSB thin film is more unfavorable for the following deposition of small NPs, resulting in less small NPs in the non-stratified thin film. What is more, these LbL thin films are relatively uniform (Figure S1, Supporting Information File 1), which is important for reproducible SERS performance [18].

**Figure 3:**
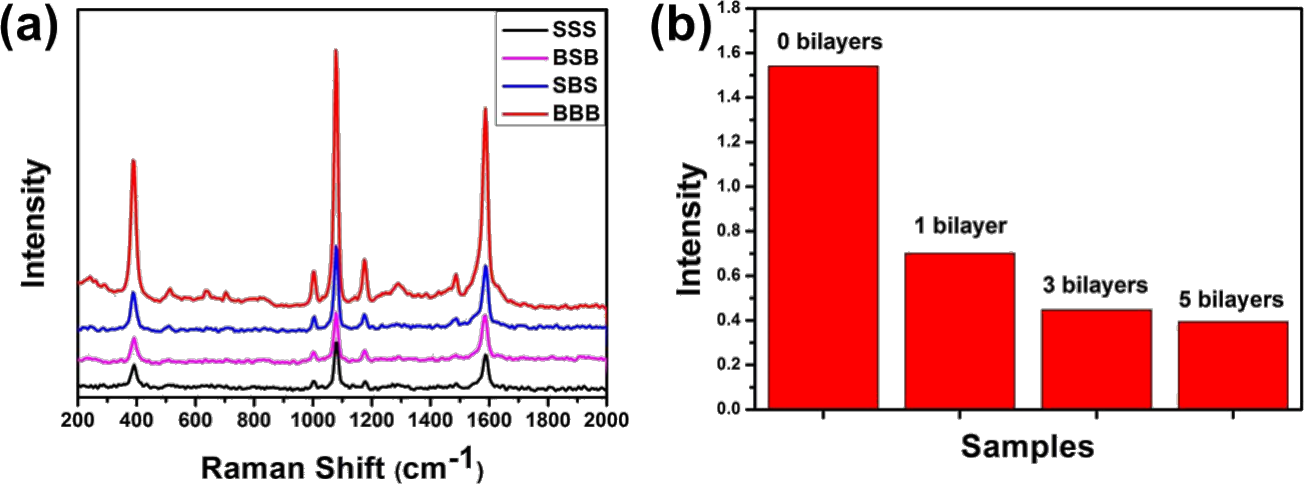
(a) SERS spectra of the sandwich-like multilayer thin films; (b) SERS intensity variations at 1078 cm^−1^ for LbL thin films inserted with different numbers of PDDA/PSS bilayers.

In order to evaluate the SERS performance of the sandwich-like nanostructures, 4-ATP was chosen as SERS probing molecule because of its well-defined Raman vibrational signatures [24]. Figure 3a shows the SERS spectra of the corresponding multilayer thin films. It is noted that the spectra are dominated by the a_1_ vibration modes, which show distinct peaks at 1587 cm^−1^ (ν_C–C_) and 1078 c^−1^ (ν_C–S_). It suggests that the electromagnetic ﬁeld enhancement dominates the SERS performance [15]. The relative SERS intensities of the films follow the order: BBB > SBS > BSB > SSS. Moreover, the actual SERS performance, which is described by the enhancement factors (EF) of these multilayer thin films, was calculated using the equation EF = (*I*_SERS_/*N*_ads_)/(*I*_bulk_/*N*_bulk_), where *I*_SERS_ and *I*_bulk_ are the intensity of a vibrational mode in the SERS spectrum and bulk sample, and *N*_ads_ and *N*_bulk_ are the number of molecules adsorbed on the SERS substrate and bulk molecules excited by the laser, respectively. Using the 1587 cm^−1^ band, the EF values for the SSS, SBS, BSB and BBB thin films are calculated to be about 5.9 × 10^4^, 5.4 × 10^4^, 5.0 × 10^4^ and 3.6 × 10^4^, respectively (see Supporting Information File 1 for the detailed calculation). It is known that SERS activity is impacted by multiple factors such as size, shape and interparticle coupling of NPs. Compared with the BBB thin film, the increase of the enhancement factor of the SSS thin film could be primarily ascribed to electromagnetic enhancement of increased NP numbers, since there might be more hot-spots in the SSS thin film [25–26]. As shown in Figure 2b and Figure 2c, more hot-spots would also be generated for the SBS thin film, resulting in a better SERS performance than the BSB thin film. Though NP size was demonstrated to greatly impact the SERS performance, it is difficult to obtain dense large NPs layer using the LbL assembly, which is critical for the improved electromagnetic enhancement.

It has been shown that the distance between adjacent NP layers has notable effects on the optical, magnetic and electrochemical properties of LbL NP thin films [27–33]. However, this interesting effect on SERS properties is almost unexplored [34]. Since the interparticle distance may strongly influence the SERS performance, PDDA/PSS bilayers were introduced to separate the neighboring NP layers. Figure 3b displays the SERS intensities at 1078 cm^−1^ for sandwich-like thin films inserted with different numbers of PDDA/PSS bilayers. Obviously, the SERS intensity decreased significantly with increasing number of separating PDDA/PSS bilayers. This is possibly because localized surface plasmon from Au NPs of neighboring layer was not coupled intensively, after the interlayer gap was broadened by inserting bilayers [35]. It is noteworthy that NP layers separated by three polyelectrolyte monolayer provided the strongest SERS signals, as recently presented by Carsten Rockstuhl and co-workers [34]. Nevertheless, the fact that the multilayer NP thin film without separating layers possesses the strongest SERS performance is reasonable, because the thickness of one polyelectrolyte monolayer assembled at high salt concentration (1.5 M NaCl, this work) may be similar to that of three polyelectrolyte monolayers assembled at low salt concentration (0.1 M NaCl, work of Rockstuhl et al.) [36]. Overall, controlling the interlayer distance of the NP layers is another efficient way to tune the SERS properties of multilayer thin films.

## Conclusion

Sandwich-like LbL assemblies of Au NPs were designed as model SERS substrate. The SERS performance could be readily tuned by using Au NPs of different sizes or introducing insert layers with controllable thickness. The methods and strategies involved in this work are rather simple. The fabricated SERS substrates may pave the way for highly efficient and sensitive sensing of small molecules.

## Supporting Information

File 1 Additional experimental data.

## References

[1] Schlücker S (2014). Angew Chem, Int Ed.

[2] Moskovits M (2013). Phys Chem Chem Phys.

[3] Pieczonka N P W, Aroca R F (2008). Chem Soc Rev.

[4] Ko H, Singamaneni S, Tsukruk V V (2008). Small.

[5] Tong L, Zhu T, Liu Z (2011). Chem Soc Rev.

[6] Jiang C, Tsukruk V V (2006). Adv Mater.

[7] Borges J, Mano J F (2014). Chem Rev.

[8] Richardson J J, Björnmalm M, Caruso F (2015). Science.

[9] Abalde-Cela S, Ho S, Rodríguez-González B, Correa-Duarte M A, Álvarez-Puebla R A, Liz-Marzán L M, Kotov N A (2009). Angew Chem, Int Ed.

[10] Bao Y, Vigderman L, Zubarev E R, Jiang C (2012). Langmuir.

[11] Tian R, Li M, Teng H, Luo H, Yan D, Wei M (2015). J Mater Chem C.

[12] Aoki P H B, Alessio P, De Saja J A, Constantino C J L (2010). J Raman Spectrosc.

[13] dos Santos D S, Goulet P J G, Pieczonka N P W, Oliveira O N, Aroca R F (2004). Langmuir.

[14] Zhang F, Srinivasan M P (2008). J Colloid Interface Sci.

[15] Hu X, Cheng W, Wang T, Wang Y, Wang E, Dong S (2005). J Phys Chem B.

[16] Koo H Y, Choi W S, Park J-H, Kim D-Y (2008). Macromol Rapid Commun.

[17] Zhang L, Wang C, Zhang Y (2012). Appl Surf Sci.

[18] Liu Z, Yan Z, Bai L (2016). Appl Surf Sci.

[19] Enustun B V, Turkevich J (1963). J Am Chem Soc.

[20] Liu Z, Chang T, Huang H, He T (2013). RSC Adv.

[21] Liu Z, Chang T, Huang H, He T (2015). ACS Appl Mater Interfaces.

[22] Ostrander J W, Mamedov A A, Kotov N A (2001). J Am Chem Soc.

[23] Yuan W, Li C M (2009). Langmuir.

[24] Huang Y-F, Wu D-Y, Zhu H-P, Zhao L-B, Liu G-K, Ren B, Tian Z-Q (2012). Phys Chem Chem Phys.

[25] Fang P-P, Li J-F, Yang Z-L, Li L-M, Ren B, Tian Z-Q (2008). J Raman Spectrosc.

[26] Joseph V, Matschulat A, Polte J, Rolf S, Emmerling F, Kneipp J (2011). J Raman Spectrosc.

[27] Schmitt J, Decher G, Dressick W J, Brandow S L, Geer R E, Shashidhar R, Calvert J M (1997). Adv Mater.

[28] Jiang G, Baba A, Ikarashi H, Xu R, Locklin J, Kashif K R, Shinbo K, Kato K, Kaneko F, Advincula R (2007). J Phys Chem C.

[29] Vial S, Pastoriza-Santos I, Pérez-Juste J, Liz-Marzán L M (2007). Langmuir.

[30] Kiel M, Mitzscherling S, Leitenberger W, Santer S, Tiersch B, Sievers T K, Möhwald H, Bargheer M (2010). Langmuir.

[31] Chaikin Y, Leader H, Popovitz-Biro R, Vaskevich A, Rubinstein I (2011). Langmuir.

[32] Pichon B P, Louet P, Felix O, Drillon M, Begin-Colin S, Decher G (2011). Chem Mater.

[33] Schmidt A R, Nguyen N D T, Leopold M C (2013). Langmuir.

[34] Mühlig S, Cialla D, Cunningham A, März A, Weber K, Bürgi T, Lederer F, Rockstuhl C (2014). J Phys Chem C.

[35] Yi Z, Yi Y, Luo J, Ye X, Wu P, Ji X, Jiang X, Yi Y, Tang Y (2015). RSC Adv.

[36] McAloney R A, Sinyor M, Dudnik V, Goh M C (2001). Langmuir.

