# Supplementary material for: Sandwich-like layer-by-layer assembly of gold nanoparticles with tunable SERS properties
Source: Beilstein J Nanotechnol. 2016 Jul 15;7:1028–32. doi: 10.3762/bjnano.7.95 (PMC4979642; doi:10.3762/bjnano.7.95)
Supplement: File 1 — Additional experimental data. [file Beilstein_J_Nanotechnol-07-1028-s001.pdf]

Supporting Information

for

Sandwich-like layer-by-layer assembly of gold nanoparticles with tunable SERS properties

Zhicheng Liu*[1], Lu Bai[2], Guizhe Zhao[1] and Yaqing Liu*[1]

Address: [1]Shanxi Province Key Laboratory of Functional Nanocomposites, School of Materials Science and Engineering, North University of China, Taiyuan 030051, China and [2]School of Chemical and Environmental Engineering, North University of China, Taiyuan 030051, China

Email: Zhicheng Liu* - zcliu11@gmail.com ; Yaqing Liu* - zfflyq98@163.com

* Corresponding author

Additional experimental data

Calculation of the EF value:

EF = $(I_{SERS}/N_{ads})/(I_{bulk}/N_{bulk})$

N_{bulk} is the number of molecules of the solid 4-ATP in the laser illumination volume. In our work, the laser spot of 100 μm in diameter and the penetration depth (ca. 180 μm) of the focused laser beam are used. Taking the density of the solid 4-ATP (1.18 g/cm^3) into account, N_{bulk} was calculated to be about 8×10^{15} molecules within the illuminated laser light.

N_{abs} can be obtained according to the method proposed by Murphy and co-workers [1]. According to the report by Kim et al., each 4-ATP molecule occupies ca. 0.20 nm^2 on the surface of Au nanoparticles [2]. Because the laser spot is about 7.85×10^{-5} cm^2, the number of the molecules on the BBB thin film within the laser spot is assumed to be 1.8×10^{11}. This is estimated by counting the number of the big Au nanoparticles on the spot and calculating the surface area of those Au nanoparticles.

Taking account of the intensity ratio of the 1587 cm^{-1} band, the the EF value for the BBB thin films are calculated to be ca. 3.6×10^4. Other EF values could be obtained similarly.

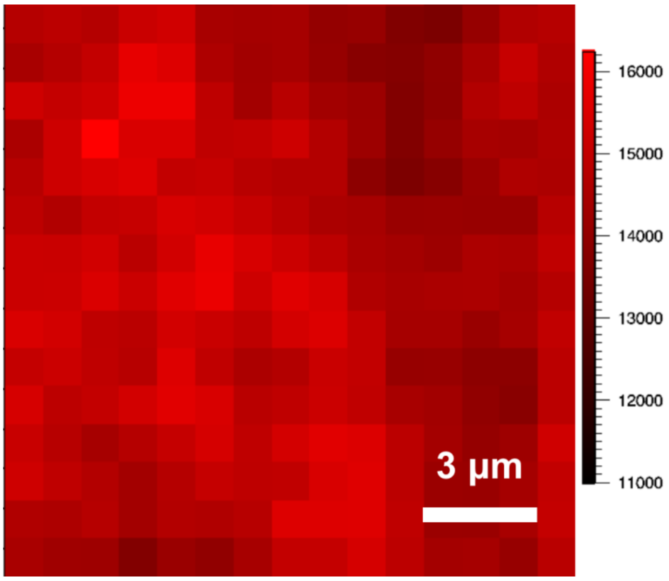

Figure S1: SERS mapping (15 × 15 μm^2, step 1 μm) of the 1078 cm^{-1} band of 4-ATP on the BBB thin film. The spectra were measured using a laser scanning confocal spectrometer (Renishaw inVia) with a 785 nm laser excitation.

References

[1] Orendorff, C. J.; Gole, A.; Sau, T. K.; Murphy, C. J. *Anal. Chem.* **2005,** *77,* 3261. doi:10.1021/ac048176x

[2] Kim, K.; Yoon, J. K. *J. Phys. Chem. B.* **2005,** *109,* 20731. doi:10.1021/jp052829b
